# Influence of nutrition on infection and re-infection with soil-transmitted helminths: a systematic review

**DOI:** 10.1186/1756-3305-7-229

**Published:** 2014-05-19

**Authors:** Peiling Yap, Jürg Utzinger, Jan Hattendorf, Peter Steinmann

**Affiliations:** 1 Department of Epidemiology and Public Health, Swiss Tropical and Public Health Institute, Basel, Switzerland; 2 University of Basel, Basel, Switzerland

**Keywords:** Soil-transmitted helminths, Nutrition, Anthelminthic treatment, Re-infection, Systematic review

## Abstract

**Background:**

The relationship between nutrition and soil-transmitted helminthiasis is complex and warrants further investigation. We conducted a systematic review examining the influence of nutrition on infection and re-infection with soil-transmitted helminths (i.e. *Ascaris lumbricoides*, hookworm, *Trichuris trichiura* and *Strongyloides stercoralis*) in humans. Emphasis was placed on the use of nutritional supplementation, alongside anthelminthic treatment, to prevent re-infection with soil-transmitted helminths.

**Methods:**

We searched eight electronic databases from inception to 31 July 2013, with no restriction of language or type of publication. For studies that met our inclusion criteria, we extracted information on the soil-transmitted helminth species, nutritional supplementation and anthelminthic treatment. Outcomes were presented in forest plots and a summary of findings (SoF) table. An evidence profile (EP) was generated by rating the evidence quality of the identified studies according to the GRADE system.

**Results:**

Fifteen studies met our inclusion criteria; eight randomised controlled trials and seven prospective cohort studies. Data on *A. lumbricoides* were available from all studies, whereas seven and six studies additionally contained data on *T. trichiura* and hookworm, respectively. None of the studies contained data on *S. stercoralis*. Positive effects of nutritional supplementation or the host’s natural nutritional status on (re-)infection with soil-transmitted helminths were reported in 14 studies, while negative effects were documented in six studies. In terms of quality, a high, low and very low quality rating was assigned to the evidence from four, six and five studies, respectively.

**Conclusions:**

Our findings suggest that the current evidence-base is weak, precluding guidelines on nutrition management as a potential supplementary tool to preventive chemotherapy targeting soil-transmitted helminthiasis. Moreover, several epidemiological, immunological and methodological issues have been identified, and these should be considered when designing future studies.

## Background

There is a Chinese proverb that goes “Let food be medicine” (translation of “以食为疗”). The traditional belief that inspired this saying is that, in addition to nutritional value, food has medicinal properties too. Today, it is well established that the consumption of appropriate nutrients is critical to build up one’s immune defense against pathogens [1]. Indeed, it has been shown that undernutrition increases the general susceptibility of an individual to viral, bacterial and parasitic infections [2-6], while infections negatively impact on the nutritional status, resulting in a vicious cycle of undernutrition and infection [7-9].

Roundworm (*Ascaris lumbricoides*), whipworm (*Trichuris trichiura*), the hookworms (*Ancylostoma duodenale* and *Necator americanus*) and threadworm (*Strongyloides stercoralis*) are collectively termed soil-transmitted helminths [10-12]. Soil-transmitted helminthiases are the most prevalent neglected tropical diseases; over 5 billion people are at risk and more than 1 billion people are currently infected [11,13-16]. The global burden attributable to these intestinal nematode infections is estimated at 5.2 million disability-adjusted life years (DALYs) [17]. Infections are concentrated in impoverished communities in tropical and sub-tropical regions of sub-Saharan Africa, Asia and Latin America, where poor personal hygiene practices are common and the provision of sanitation and clean water is deficient [15,16,18-20]. The World Health Organization (WHO) advocates preventive chemotherapy, the regular administration of anthelminthic drugs to at-risk populations, to control the morbidity due to soil-transmitted helminthiasis [21,22]. However, re-infection occurs rapidly, especially in the absence of targeted hygiene education and measures to improve sanitation and water supply [20,23-25].

**Figure 1:**
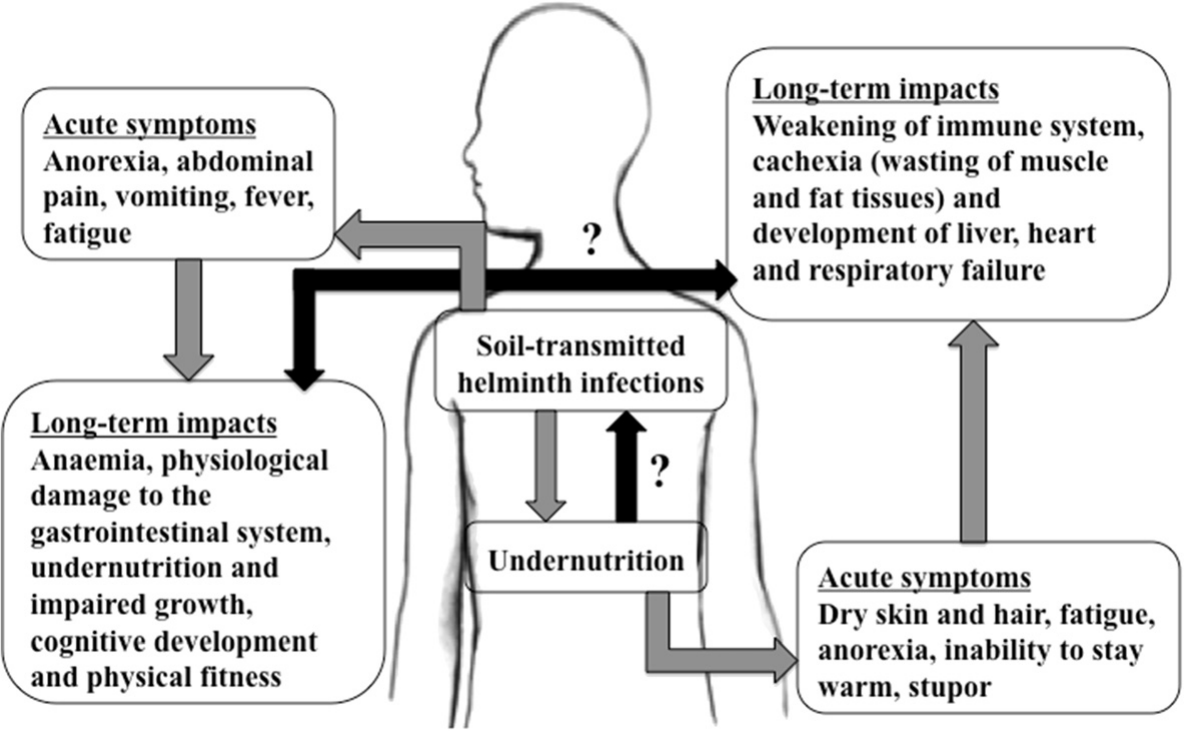
**Conceptual framework underpinning this systematic review.** The black arrows indicate research gaps in the understanding of the interactions between soil-transmitted helminth infections and undernutrition.

In the context of the systematic review presented here, undernutrition is defined as the outcome of insufficient intake, or failure of the body to absorb, one or more essential macro- or micronutrients [3]. According to the Food and Agriculture Organization (FAO) of the United Nations (UN), the global number of undernourished people stood at approximately 870 million in the years 2010 to 2012. The majority of them lived in sub-Saharan Africa, Southern Asia and the Caribbean [26]. Given the geographical overlap with areas where the highest prevalences of soil-transmitted helminth infections are found, it is not surprising that a considerable fraction of undernourished people are simultaneously infected with soil-transmitted helminths. As illustrated in Figure 1, an individual harbouring soil-transmitted helminths might experience anaemia, physiological damage to the gastrointestinal system and other symptoms, which in turn can exacerbate nutritional deficiencies. It is commonly accepted that this could ultimately lead to impaired growth and cognitive development [27-31]. However, findings from studies pertaining to undernutrition and the resulting susceptibility to soil-transmitted helminthiasis are inconclusive, and the true cause-effect relationship, as well as the magnitude of any causal link, remains to be investigated [32].

To address both undernutrition and soil-transmitted helminthiasis, a dual intervention approach has been proposed [5]. Nutritional supplementation is commonly employed to address undernutrition and has been proven to be effective in reducing nutritional deficiencies [33]. However, nutrition management, alongside anthelminthic treatments and with an aim to reduce re-infection with soil-transmitted helminths, remains controversial, and its practical significance and public health potential have yet to be fully explored [32]. Given that one of the Millennium Development Goals (MDGs) is to “halve, between 1990 and 2015, the proportion of people who suffer from hunger” [34], the role of nutrition management on the (re-)infection with soil-transmitted helminths should be investigated. This systematic review summarises the available evidence on influence of nutrition on (re-)infection with soil-transmitted helminths in humans, with a focus on the use of nutritional supplementation, provided together with anthelminthic drugs, to reduce (re-)infection.

## Methods

### Criteria for considering studies for this review

#### ***Types of studies***

We included randomised controlled trials and prospective cohort studies.

#### ***Types of outcome measures***

Primary outcomes for single and concurrent infections with soil-transmitted helminths were: (i) (re-)infection rate described as (changes in) prevalence (%); and (ii) (changes in) infection intensity in terms of (changes in) eggs per 1 g of stool (EPG). Secondary outcomes were: (i) immune responses to nutritional supplementation that is relevant for soil-transmitted helminth infections; and (ii) other health benefits of nutritional supplementation. Reported detrimental effects of nutritional supplementation in study participants were recorded as adverse outcomes.

#### ***Types of comparisons***

The following comparisons were included in this review: (i) macro-nutrient supplementation *versus* placebo; (ii) multi-micronutrient supplementation *versus* placebo; (iii) single micronutrient supplementation *versus* placebo; and (iv) undernourished hosts *versus* well-nourished hosts (in their natural states).

#### ***Exclusion criteria***

Cross-sectional or case–control studies that compared the nutrition status or growth of infected *versus* non-infected hosts (in their natural states or treated with anthelminthics) were excluded for their lack of potential to demonstrate causality. Reviews and studies that focused on interactions between malnutrition and immunity in general, with no specific reference to soil-transmitted helminths, were also excluded. Lastly, case reports or case series were also excluded.

### Search methods for identification of studies

We searched eight readily available electronic databases: (i) PubMed/Medline; (ii) Embase; (iii) Cochrane Library; (iv) Cochrane Central Register of Controlled Trials (CENTRAL); (v) Virtual Health Library (VHL); (vi) Science Direct; (vii) China National Knowledge Infrastructure (CNKI); and (viii) VIP Information, from inception to 31 July 2013. No language or publication type restrictions were applied. The following keywords and combinations thereof were used: “reinfection”, “soil-transmitted helminths”, “multiparasitism”, “polyparasitism”, “infection intensity”, “hookworm”, “*Ascaris*”, “*Strongyloides*” “*Trichuris*”, “nutrition”, “undernutrition”, “malnutrition”, “iron”, “zinc”, “vitamin”, “nutrition supplementation” and “micronutrient supplementation”. The detailed search strategies are described in Additional file 1: Table S1. Bibliographies of identified studies were checked manually for potential additional references.

### Data collection and analysis

#### ***Selection of studies***

The study selection process was performed by the first author. Potentially relevant studies were identified by screening the titles and, if available, the abstracts of publications. Full manuscript texts were assessed if the relevance of a publication could not be judged from its title or abstract. Finally, the full texts of all potentially relevant studies were evaluated against the predefined inclusion criteria.

#### ***Data extraction***

The full text of all selected studies was reviewed and information on the study period, location, design, objectives, method of recruitment, sample size, and socio-demographic characteristics of, and inclusion criteria for, study participants were extracted. In addition, information on the soil-transmitted helminth species and nutritional supplementation used (type, duration, dosage, frequency, type of control group and presence of co-interventions, if any) were gathered, along with both primary and secondary outcomes.

#### ***Data analysis***

Where relevant data were available, unadjusted odds ratios (ORs) with 95% confidence intervals (CIs), comparing prevalence rates among individuals who received nutritional supplementation/were well-nourished, and those who received placebo/were under-nourished, were calculated. Fixed-effects meta-analyses were conducted for *A. lumbricoides*, *T. trichiura*, hookworm and, when the results were not species-specific, soil-transmitted helminths combined. No studies were identified for *S. stercoralis*, precluding any meta-analysis. Unweighted ORs were pooled according to the type of nutritional supplementation/natural nutrition status of the host and an overall OR was also generated. An OR of less than 1.0 indicates a decrease in the odds of being (re-)infected with soil-transmitted helminths among individuals who received nutritional supplementation/were well-nourished. All statistical analyses were performed with STATA version 10.0 (STATA Corp.; College Station, TX, USA).

In addition, qualitative content analysis was conducted for all studies included in the systematic review. Individual outcome measures reported in the articles were summarised and categorised into “strong positive effect”, “moderate-to-weak positive effect”, and “negative effect”. Outcome measures with a *P*–value <0.05 were considered to be of statistical significance. The categories were defined as: (i) “strong positive effect” if the intervention/well-nourished group showed a more than two-fold and statistically significant improvement compared to the control/undernourished group; (ii) “moderate-to-weak positive effect” if the improvements were between nil and two-fold or more than two-fold but not statistically significant; and (iii) “negative effect” whenever outcome measures worsened in the intervention/well-nourished group as compared to the control/undernourished group. Consequently, an article could have outcome measures listed in different categories if it contained results of different effect size, e.g. for different soil-transmitted helminth species or different interventions.

#### ***Quality assessment of included studies***

Following the GRADE rating system [35], outcomes of each study were checked for consistency, precision, directness and the magnitude of the observed effect. The risk of bias was assessed by examining the method of allocation to different study groups; whether randomisation, concealment (protection of the randomisation process to ensure treatment allocation is not known prior to study initiation) or blinding of participants, providers and outcome assessors to the type of intervention received, were performed; the presence of selection, measurement and reporting bias; and loss to follow-up. The evidence quality of the identified studies was rated as high, moderate, low or very low, according to the GRADE guidelines. The full review protocol is available from the corresponding author.

## Review

### Characteristics of studies

**Figure 2:**
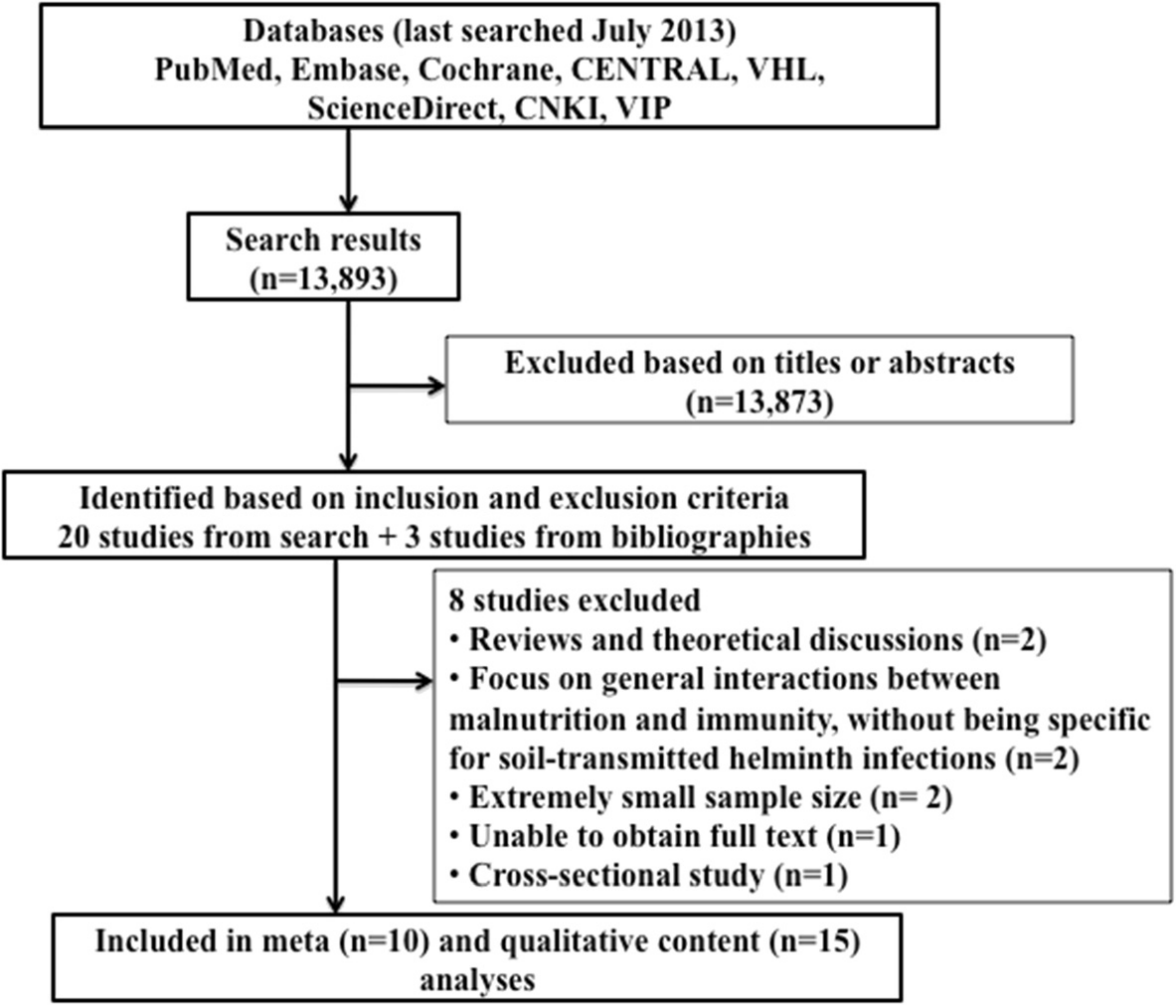
Search strategy for the identification of studies examining the influence of nutrition on (re-)infection with soil-transmitted helminths in humans.

The literature searches yielded a total of 13,893 hits (Figure 2). Based on the titles or abstracts, only 20 studies were identified for inclusion. The full texts of all identified studies were retrieved and assessed. Screening of the bibliographies of these studies revealed an additional three studies. Among these 23 studies, eight were excluded (cross-sectional survey, n = 1; reviews, n = 2; interactions between malnutrition and immunity with no specific reference to soil-transmitted helminths, n = 2; small case series of 3–12 individuals, n = 2; and unable to obtain the full text, n = 1) (Additional file 2: Table S2).

Among the 15 studies included in our final analysis, six had been conducted in Central America [36-41]; four in Africa [42-45]; three in Asia [46-48]; and two in South America [49,50]. These studies were all published in English.

Characteristics of the 15 studies are listed in the summary of findings (SoF) table (Table 1 and Additional file 3: Table S3). Eight studies were randomised controlled trials; two of the publications [47,48] actually contained data from the same randomised controlled trial, with different components of the findings published in each of them. In addition, there were seven prospective cohort studies. Data pertaining to *A. lumbricoides* were available from each study, while seven studies additionally reported data on *T. trichiura* and six on hookworm. None of the studies identified contained data on *S. stercoralis*. Fourteen studies included children only, aged between 5 months and 15 years. One study [43] focused on adolescents and adults (age range: 16–63 years). The design of seven studies included the administration of albendazole or mebendazole to participants at baseline and monitoring subjects for re-infection with soil-transmitted helminths over a period of 4 to 15 months, during which regular nutritional supplementation or placebo was provided. Nutritional supplementation included multi-micronutrient biscuits or tablets, vitamin A given as retinol solution, zinc administered as zinc methionine solution or iron taken as ferrous dextran tablets. In two studies [38,39], young children, aged 5–15 months, were given only nutritional supplementation or placebo, without preventive chemotherapy, and followed for 12–15 months for *A. lumbricoides* infection or immune responses against this nematode. Finally, six studies evaluated albendazole, mebendazole or oxantel/pyrantel treatments with evaluation of the participants, stratified by their natural nutrition states, occurring 8 months [50] or 12 months [49] after the treatment period. In another study, albendazole was administered to children who were followed, in their natural nutrition states, over two treatment and re-infection cycles [41].

**Table 1:** Summary of findings from 15 studies meeting the inclusion criteria of the systematic review pertaining to undernutrition and re-infection with soil-transmitted helminths

**Characteristics of studies**			
**Study design**^**a**^**; location; sample size; intestinal helminth examined**^**b**^	**Study subjects**	**Intervention/control arms**	**Outcomes**
1) Nga *et al.* 2011 [48]; factorial RCT; Vietnam; N = 466; A, T, H	6- to 8-year-old children	i) Multi-micronutrient biscuits fortified with iron, zinc, iodine and vitamin A given on 5 days per week for 4 months. Albendazole (400 mg, single dose) at baseline	**Primary:** i) Moderate-to-weak positive effect on the reduction of the infection intensity of all intestinal helminth species in children taking 'fortified biscuits and albendazole’ *versus* 'albendazole alone’
		ii) Multi-micronutrient biscuits and placebo, identical looking to albendazole	**Secondary:** Moderate-to-weak positive effect on growth and cognition in children receiving fortified biscuits
		iii) Non-fortified, identical looking biscuits and albendazole at baseline	
		iv) Non-fortified, identical looking biscuits and albendazole placebo	
2) Nga *et al.* 2009 [47]; factorial RCT; Vietnam; N = 466; A, T, H	6- to 8-year-old children	i) Multi-micronutrient biscuits fortified with iron, zinc, iodine and vitamin A given on 5 days per week for 4 months. Albendazole (400 mg, single dose) at baseline	**Primary:** i) Strong positive effect on the reduction of the prevalence of all intestinal helminth species in children taking 'fortified biscuits and albendazole’ *versus* 'albendazole alone’
		ii) Multi-micronutrient biscuits and placebo, identical looking to albendazole	**Secondary:** Strong positive effect on reducing anaemia, zinc and iodine deficiencies in children receiving fortified biscuits
		iii) Non-fortified, identical looking biscuits and albendazole at baseline	
		iv) Non-fortified, identical looking biscuits and albendazole placebo	
3) Nchito *et al.* 2009 [45]; factorial RCT; Zambia; N = 215; A	7- to 15-year-old children	i) Albendazole (400 mg) given to all study participants on 2 consecutive days at baseline	**Primary:** i) Moderate-to-weak effects on the reduction of the prevalence of *A. lumbricoides* in children taking 'iron only’ and 'multi-micronutrients only’ *versus* 'placebo’. Negative effect on the reduction of the prevalence of *A. lumbricoides* in children taking 'iron with multi-micronutrients’ *versus* 'placebo’
		ii) Multi-micronutrient tablet fortified with vitamin A, B_1_, B_2_, B_6_, B_12_, C, D and E, niacin, folic acid, zinc, iodine, copper and selenium every school day for 10 months. Ferrous dextran tablet (equivalent to 60 mg of elemental iron) every school day for 10 months	ii) Moderate-to-weak positive effect on the reduction of the infection intensity of *A. lumbricoides* in children taking 'iron only’ *versus* 'placebo’. Negative effects on the reduction of the infection intensity of *A. lumbricoides* in children taking 'iron with multi-micronutrients’ and 'multi-micronutrients only’ *versus* 'placebo’
		iii) Multi-micronutrient tablet and placebo iron tablet	
		iv) Placebo multi-micronutrient tablet and ferrous dextran tablet	
		v) Placebo multi-micronutrient tablet and placebo iron tablet	
4) Long *et al.* 2007 [39]; factorial RCT; Mexico; N = 707; A	6- to 15-month-old children	i) Vitamin A (given as 20,000 IU of retinol for children <1 year and 45,000 IU for children >1 year) every 2 months for 1 year. Zinc methionine (equivalent to 20 mg of elemental zinc)	**Primary:** i) Strong positive effect on the reduction of the prevalence of *A. lumbricoides* in children taking 'zinc alone’ *versus* 'placebo’. Negative effects on the reduction of the prevalence of *A. lumbricoides* in children taking **'**vitamin A with zinc’ and 'vitamin A alone’ *versus* placebo
		ii) Zinc methionine only	**Secondary:** i) A combination of vitamin A and zinc had a moderate-to-weak positive effect on the reduction of *A. lumbricoides* infection duration and a strong positive effect on the reduction of *A. lumbricoides*-associated diarrhoea
		iii) Vitamin A only	
		iv) Placebo	
5) Long *et al.* 2006 [38]; RCT; Mexico; N = 127; A	5- to 15-month-old children	i) Vitamin A (given as 20,000 IU of retinol for children <1 year and 45,000 IU for children >1 year) every 2 months for 15 months	**Secondary:** i) Strong positive effect on the increase of interleukin 4 (IL-4) levels in vitamin A supplemented children *versus* placebo
		ii) Placebo	
6) Olsen *et al.* 2003 [44]; RCT; Kenya; N = 977; A, T, H	8- to 18-year-old children	i) Albendazole (600 mg, single dose) given to all children at baseline and 4 weeks after baseline (600 mg, single dose) if child was still infected	**Primary:** i) For children taking 'multi-micronutrients’ *versus* 'placebo’, moderate-to-weak positive effects on the reduction of the prevalence of *A. lumbricoides* and hookworm and negative effect on the reduction of the prevalence of *T. trichiura*
		ii) Multi-micronutrient tablet fortified with vitamin A, B_1_, B_2_, B_6_, B_12_, C, D and E, niacin, folic acid, zinc, iodine, copper, iron and selenium every school day for 11 months	ii) For children taking 'multi-micronutrients’ *versus* 'placebo’, moderate-to-weak positive effects on the reduction of the infection intensity of all intestinal helminth species
		iii) Placebo, identical looking to the multi-micronutrient tablet	
7) Olsen *et al.* 2000 [43]; RCT; Kenya; N = 329; A, T, H	4- to 15-year-old children (n = 200) and 16- to 63-year-old adolescents and adults (n = 129)	i) Albendazole (400 mg, once a day for 3 consecutive days) at baseline for all individuals and if any individual was still infected between 3 and 6 months after baseline, re-treatment (400 mg, single dose) was given	**Primary:** i) For children taking 'iron’ *versus* 'placebo’, moderate-to-weak positive effects on the reduction of the prevalence of all intestinal helminth species and in the reduction of infection intensity of hookworm. Negative effects on the reduction of infection intensity of *A. lumbricoides* and *T. trichiura*
		ii) Ferrous dextran tablet (equivalent to 60 mg of elemental iron) twice weekly for 12 months	ii) For adolescents/adults taking 'iron’ *versus* 'placebo’, strong positive effects on the reduction of the prevalence of *A. lumbricoides* and *T. trichiura* and moderate-to-weak positive effect on the reduction of the prevalence of hookworm. In terms of infection intensity reduction, negative effects for *A. lumbricoides* and *T. trichiura* and moderate-to-weak positive effect for hookworm
		iii) Placebo identical looking to the ferrous dextran tablet	
8) Grazioso *et al.* 1993 [36]; RCT; Guatemala; N = 130; A, T	65- to 95-month-old children	i) Mebendazole (100 mg twice daily for 3 days) at baseline for all individuals	**Primary:** i) Negative effect on the reduction of the prevalence of *A. lumbricoides* and *T. trichiura* (mentioned collectively) in children taking 'zinc’ *versus* 'placebo’
		ii) Tablet containing zinc (10 mg) and vitamin A, E, C, B_6_, B_12_ and D, folic acid, thiamin, riboflavin, niacinamide, pantothenic acid, iron, copper, iodine, selenium, chromium and magnesium given on every school day for 120–150 days	
		iii) Different colour-coded tablets, containing all the micronutrients, except for zinc, found in the intervention tablet	
9) Halpenny *et al.* 2013 [41]; CP; Panama; N = 87-279; A, H	<5-year-old children	i) Cycle 1: albendazole (200–400 mg depending on age, single dose) to all children >12 months at baseline. Children followed up for 9 months	**Primary:** i) Strong positive effect on the reduction of *A. lumbricoides* infection intensity at the end of cycle 1 in children with higher height-for-age (HAZ) *versus* their peers with lower HAZ score ii) Strong positive effect on the reduction of hookworm infection intensity at the end of cycle 2 in children with higher height-for-age (HAZ) *versus* their peers with lower HAZ score
		ii) Cycle 2: albendazole (200–400 mg depending on age, single dose) to all children >12 months at baseline. Children who remained infected with at least 1 soil-transmitted helminth were given another single dose of albendazole. Children followed up for 6 months	
10) Hesham Al-Mekhlafi *et al.* 2008 [46]; CP; Malaysia; N = 120; A, T, H	7- to 12-year-old children	i) Albendazole (400 mg, once a day for 3 consecutive days) for all children at baseline. Children followed for 6 months to investigate predictors of re-infection	**Primary:** i) Moderate-to-positive effects on the reduction of the prevalence of *A. lumbricoides*, *T. trichiura* and hookworm (mentioned collectively) in non-stunted children *versus* stunted children
11) Payne *et al*. 2007 [40]; CP; Panama; N = 328; A	12- to 60-month-old children	i) One-off supplementation with vitamin A (60 mg retinol) given by the Ministry of Health	**Primary:** i) Moderate-to-weak positive effect on the reduction of the *A. lumbricoides* prevalence and infection intensity in vitamin A-supplemented children *versus* non-supplemented ones
		ii) Albendazole (400 mg, single dose) for all children at baseline. Children were followed at 3 and 5 months post-treatment	
12) Saldiva *et al.* 2002 [37]; CP; Brazil; N = 585; A, T	1- to 10-year-old children	i) Mebendazole (triple doses at baseline and repeated 15 days after). Children were followed at 1 year post-treatment	**Primary:** i) Moderate-to-weak positive effect on the reduction of the prevalence of *A. lumbricoides* and *T. trichiura* (mentioned collectively with other helminths, such as *Hymenolepsis nana* and *S. stercoralis*) in eutrophic children *versus* undernourished children
13) Hagel *et al.* 1999 [50]; CP; Venezuela; N = 85; A	6- to 11-year-old children	i) Oxantel/pyrantel (20 mg/kg) monthly for 12 months for all children. Children were followed at 8 months after the 12 months of treatment	**Primary:** i) Strong positive effect on the reduction of the prevalence of *A. lumbricoides* in children >10th percentile for height *versus* children ≤10th percentile for height
14) Kightlinger *et al.* 1996 [42]; CP; Madagascar; N = 360-619; A	4- to 10-year-old children	i) Mebendazole (500 mg, single dose) was given to all children at baseline. Children were followed at the end of 12 months, when they were given pyrantel pamoate (11 mg/kg) and 48-hour worm expulsions were performed	**Primary:** i) Moderate-to-weak positive effect on the reduction of the infection intensity of *A. lumbricoides* in the best-nourished children *versus* children with reduced growth indicators
15) Hagel *et al.* 1995 [49]; CP; Venezuela; N = 85; A	6- to 11-year-old children	i) Oxantel/pyrantel (20 mg/kg) monthly for 12 months for all children. Children were followed at the end of the 12 months of treatment	**Secondary:** i) Strong positive effect on an increase of anti-*Ascaris* IgE levels in well-nourished children *versus* under-nourished children

^a^CP, cohort prospective; RCT, randomised controlled trial.

^b^A, *A. lumbricoides*; H, hookworm; T, *T. trichiura*.

### Quality of evidence in included studies

Among the 15 studies included in this review, only four contained evidence graded as high quality (i.e. no serious limitation, inconsistency, indirectness and imprecision were present and no risk of bias was detected). No study had evidence of moderate quality. Evidence in six studies was given a low quality rating and evidence from another five studies was determined to be of very low quality (Table 2). When stratified according to the impact of the outcome measures, the eight studies with strong positive impact contained three (27%) high, four (45%) low and one (27%) very low quality evidence; the nine studies with moderate-to-weak positive effect were made up of two (22%) high, four (44%) low and three (33%) very low quality pieces of evidence; and the six studies with negative effect included one (17%) high, four (67%) low and one (17%) very low quality evidence items.

**Table 2:** GRADE evidence profile (EP) for the 15 studies included in the systematic review

	**Quality assessment of evidence**					
**Study**	**Limitation**	**Inconsistency**	**Indirectness**	**Imprecision**	**Risk of bias**	**Quality grading**
1) Nga *et al.* 2011 [48]	No serious limitation	No serious inconsistency	No serious indirectness	No serious imprecision	None detected	⊕ ⊕ ⊕⊕ High
2) Nga *et al.* 2009 [47]	No serious limitation	No serious inconsistency	No serious indirectness	No serious imprecision	None detected	⊕ ⊕ ⊕ ⊕ High
3) Nchito *et al.* 2009 [45]	Serious limitation (sample size used for analysis was smaller than that required for statistical significance; mean number of supplementation tablets taken was only 50% of tablets provided)	Serious inconsistency (administration of albendazole at baseline was not stated under study design but was mentioned under results)	No serious indirectness	No serious imprecision	Serious risk of bias (47% of children were lost to follow-up; method of recruitment and inclusion/exclusion criteria were not mentioned)	⊕ ⊕ ◯ ◯ Low
4) Long *et al.* 2007 [39]	No serious limitation	No serious inconsistency	No serious indirectness	No serious imprecision	None detected	⊕ ⊕ ⊕ ⊕ High
5) Long *et al.* 2006 [38]	No serious limitation	No serious inconsistency	No serious indirectness	No serious imprecision	None detected	⊕ ⊕ ⊕ ⊕ High
6) Olsen *et al.* 2003 [44]	Serious limitation (compliance rates for the multi-micronutrient tablet and placebo were low at 46%)	Serious inconsistency (the allocation of anthelminthic treatment and placebo was not clear)	No serious indirectness	No serious imprecision	Serious risk of bias (number of stool samples collected for each child varied)	⊕ ⊕ ◯ ◯ Low
7) Olsen *et al.* 2000 [43]	No serious limitation	Serious inconsistency (reporting of results was not consistent for all helminth species)	No serious indirectness	No serious imprecision	Serious risk of bias (method of recruitment and blinding procedures not mentioned; number of stool samples collected varied at each follow-up)	⊕ ⊕ ◯ ◯ Low
8) Grazioso *et al.* 1993 [36]	No serious limitation	Serious inconsistency (data reported under abstract is different from that found in the results section)	No serious indirectness	Serious imprecision (stratification of results according to soil-transmitted helminth species was not performed)	Very serious risk of bias (number of intervention days not clear; reporting of primary outcome measures were not complete)	⊕ ◯ ◯ ◯ Very low
9) Halpenny *et al. *2013 [41]	Serious limitation (compliance rate for albendazole at both treatment cycles was low at 48%)	No serious inconsistency	No serious indirectness	No serious imprecision	None detected	⊕ ⊕ ◯ ◯ Low
10) Hesham Al-Mekhlafi *et al.* 2008 [46]	No serious limitation	Serious inconsistency (reporting of sample size was not consistent throughout the study)	No serious indirectness	Serious imprecision (stratification of results according to soil-transmitted helminth species was not performed)	None detected	⊕ ◯ ◯ ◯ Very low
11) Payne *et al.* 2007 [40]	No serious limitation	No serious inconsistency	No serious indirectness	No serious imprecision	Serious risk of bias (vitamin A supplemented children came from families with significantly higher income and latrine access than the non-supplemented children; 34% children were lost to follow-up)	⊕ ⊕ ◯ ◯ Low
12) Saldiva *et al.* 2002 [37]	No serious limitation	Serious inconsistency (stratification of undernourished and eutrophic children not clear)	No serious indirectness	Serious imprecision (stratification of results according to soil-transmitted helminth species was not performed)	None detected	⊕ ◯ ◯ ◯ Very low
13) Hagel *et al.* 1999 [50]	No serious limitation	No serious inconsistency	No serious indirectness	No serious imprecision	Serious risk of bias (poverty level as a confounding factor was not taken into account during data analysis)	⊕ ◯ ◯ ◯ Very low
14) Kightlinger *et al.* 1996 [42]	No serious limitation	Serious inconsistency (number of children included for analysis varied for different outcome measures)	No serious indirectness	No serious imprecision	Serious risk of bias (about 41% children were lost to follow-up)	⊕ ◯ ◯ ◯ Very low
15) Hagel *et al.* 1995 [49]	No serious limitation	No serious inconsistency	No serious indirectness	No serious imprecision	None detected	⊕ ⊕ ◯ ◯ Low

### Influence of nutrition on soil-transmitted helminth infections

#### ***Multi-micronutrients***

**Figure 3:**
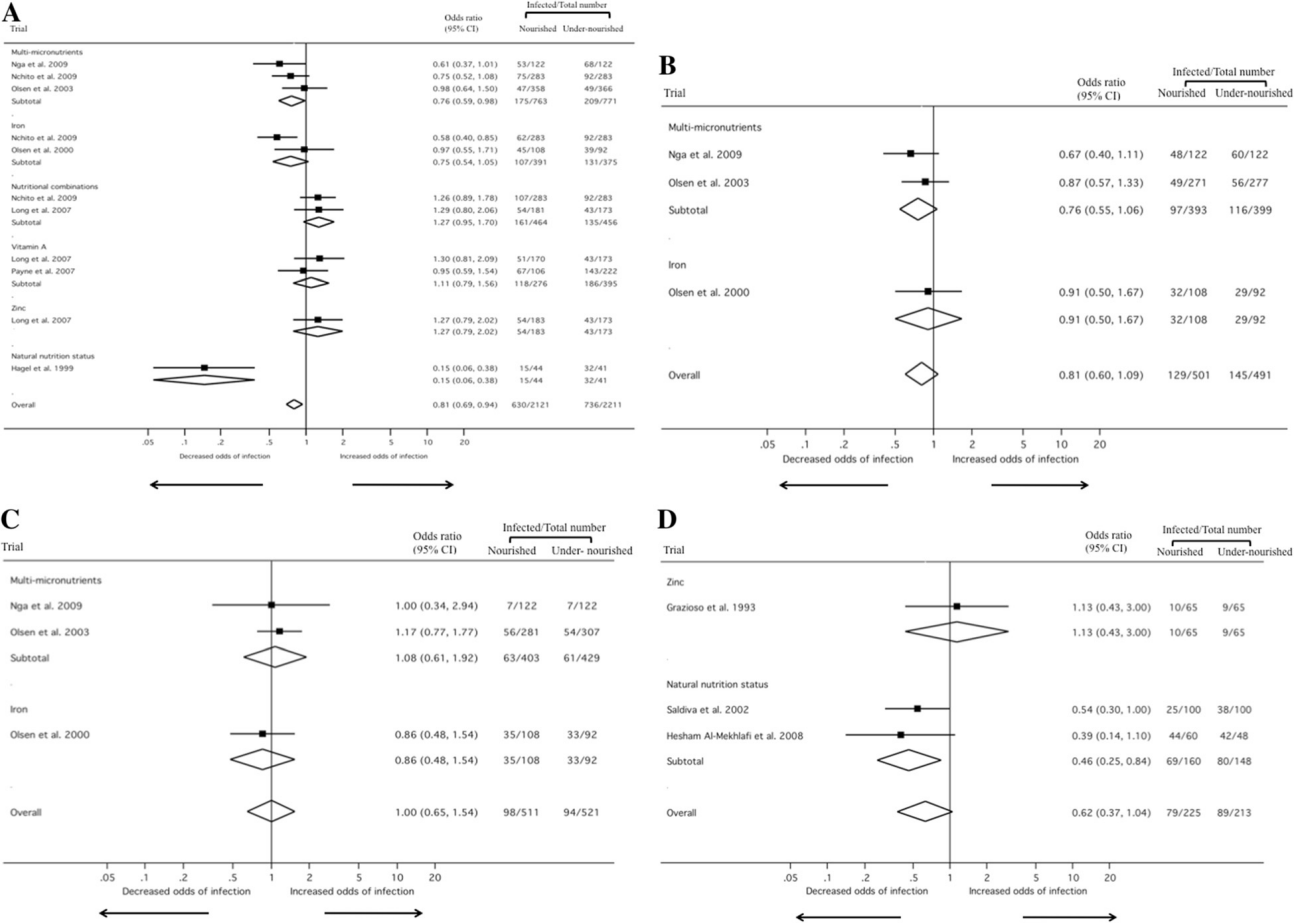
**Meta-analysis examining the association of nutritional supplementation/host’s natural nutrition status with *A. lumbricoides *** **(A), *T. trichiura *** **(B), hookworm (C) and soil-transmitted helminths combined (D) (Note: no data were identified for *S. stercoralis*).**

Multi-micronutrients, taken in the form of biscuits or tablets, lowered the re-infection rate with *A. lumbricoides* (OR: 0.76, 95% CI: 0.59-0.98, Figure 3A) and *T. trichiura* (OR: 0.76, 95% CI: 0.55-1.06, Figure 3B) but the decline failed to reach statistical significance [44,45,47]; the quality of this evidence is moderate. Slightly higher re-infection rates were observed for hookworm in subjects given micronutrients, again without statistical significance (OR: 1.08, 95% CI: 0.61-1.92, Figure 3C). The quality of this evidence is moderate. Regarding re-infection intensity, two studies containing moderate quality evidence reporting moderate-to-weak positive effects were identified for all three intestinal helminth species (1,443 participants) [44,48], and one study of low quality evidence reporting negative effects was identified for *A. lumbricoides* (215 participants) [45]. In terms of secondary outcome measures, multi-micronutrients had a strong positive effect on reducing the odds of anaemia, zinc and iodine deficiencies, but only a moderate-to-weak positive effect on growth and cognition in one study (466 participants, high quality evidence) [47,48].

#### ***Iron***

Iron tablets probably decrease the re-infection rate with *A. lumbricoides* (OR: 0.75, 95% CI: 0.54-1.05, Figure 3A), but the quality of this evidence is low [43,45]. Iron might also reduce the *A. lumbricoides* infection intensity (two studies reporting moderate-to-weak positive effects, 544 participants, low quality evidence) [43,45]. It is not clear what effect iron has on the re-infection rate and infection intensity with *T. trichiura* and hookworm, as only one study with moderate-to-weak and negative effects (329 participants, low quality evidence) was identified [43].

#### ***Vitamin A***

Vitamin A supplementation showed no effect on re-infection with *A. lumbricoides* (OR: 1.11, 95% CI: 0.79-1.56, Figure 3A; quality of evidence is moderate) [39,40]. With regards to a reduction of *A. lumbricoides* infection intensity, only one low-quality evidence with moderate-to-weak positive effects was found (328 participants) [40]. We identified no studies examining the impact of vitamin A on *T. trichiura* and hookworm re-infection. In terms of secondary outcome measures, vitamin A increased the odds of having improved levels of IL-4, which is part of the Th2 response against helminths, in one study (strong positive effect, 127 participants, high quality evidence) [38].

#### ***Zinc***

Zinc might have a negative effect on the *A. lumbricoides* re-infection rate as one study (707 participants, high quality evidence) [39] showed an increase in the prevalence of this parasite in children who took zinc tablets and another study (130 participants, very low quality evidence) [36] also found that zinc-supplemented children fared worse than their placebo counterparts in terms of soil-transmitted helminth re-infection (*A. lumbricoides* and *T. trichiura* were treated collectively and no stratification of results is available in the published article; Figure 3D). No other studies investigating the impact of zinc on *T. trichiura* and hookworm re-infection were identified.

#### ***Nutritional combinations***

Nutritional combination therapies might slightly increase the *A. lumbricoides* re-infection rate. We identified two studies, one combining iron and multi-micronutrients (OR: 1.26, 95% CI: 0.89-1.78, Figure 3A) [45], and the second, vitamin A plus zinc (OR: 1.29, 95% CI: 0.80-2.06, Figure 3A) [39]. In terms of secondary outcomes, another study reported that a combination of zinc and vitamin A reduced the mean duration of *A. lumbricoides* infections (moderate-to-weak positive effect) and lowered the incidence of *A. lumbricoides*-associated diarrhoea (strong positive effect; 707 participants, high quality evidence) [39].

#### ***Natural nutrition status of the host***

The natural nutrition status of the host might have an impact on soil-transmitted helminth re-infection according to one low-quality evidence which showed strong positive effects (279 participants; Figure 3D) [37,41,46], and four very low-quality evidences (one with strong positive and three with moderate-to-weak positive effects, 1,409 participants) [42,50]. Indeed, these five studies found that children considered to be well nourished had lower soil-transmitted helminth re-infection rates and intensities compared to their under-nourished peers. In terms of secondary outcome measures, hosts with a better nutrition status were found to have higher levels of anti-*Ascaris* IgE levels (strong positive effect) than those with poor nutrition indicators in one study (85 participants, low quality evidence) [49].

## Discussion

There is a considerable body of literature on the effects of macro- and micronutrients on host immune function and their association with infectious diseases [2,5,6,8,32,51]. However, little is known whether nutritional deficiencies have an effect on the susceptibility to infection and re-infection with soil-transmitted helminths. To fill this gap, we conducted a systematic review examining the influence of nutrition on infection and re-infection with soil-transmitted helminths. We focused particularly on the use of nutritional supplementation to prevent re-infection following anthelminthic treatment. The results from our systematic review indicate that, first, only few studies are available and, second, most of the evidence on the effects of nutritional supplementation and undernutrition on (re-)infection with soil-transmitted helminths is of low quality. Among the various nutritional supplementation interventions reviewed, multi-micronutrient supplementation seemed to have the clearest effect in terms of lowering re-infection rates and intensity of soil-transmitted helminths, whereas consumption of zinc or vitamin A alone might have a negative effect on these two outcome measures. With regard to the natural nutrition status of the host, the general trend observed was that individuals with poor nutrition indicators experienced higher re-infection rates and intensities than their well-nourished counterparts. However, the risk of confounding is higher in studies focusing on the natural nutrition status rather than controlled supplementation following randomisation. Hence, findings from the former studies have to be interpreted with care.

The evidence reported here must be seen in conjunction with the strengths and limitations of our systematic review. In terms of strength, we conducted a broad search including eight major electronic scientific literature databases that were systematically reviewed for relevant articles, complemented with hand-searches of bibliographies of identified articles. The reporting of the review was done based on the PRISMA guidelines (Additional file 4: Table S4) [52], while the GRADE system [35] was adopted for grading the quality of the reported evidence. A combination of meta-analysis and qualitative content analysis was adopted to ensure a comprehensive review. Due to the small number of studies identified, most of which with low quality evidence that is statistically insignificant, the unweighted pooled ORs should be interpreted with caution. Therefore, it is also statistically irrelevant to detect heterogeneity with Moran’s *I*^*2*^ or publication bias with the Egger’s test. However, a potential publication bias was noted based on the forest plots, where studies with smaller sample sizes presented more significant results than studies with larger sample sizes. Finally, no “grey literature” and experts’ opinions were included as the quality and strength of evidence of these sources is usually lower than that of articles published in the peer-reviewed literature.

There are several shortcomings in the included studies. None of the studies investigated the effect of nutritious whole foods as an intervention. Nutrients delivered by whole foods derived from the biological environment of the study population might have a distinct impact compared to synthetic supplements, since consumption of a broad range of nutrients from their natural sources might aid in their absorption and assimilation into the body [53]. Earlier work [54,55] did attempt to improve on whole diets and assess their impact on hookworm infection but the ill-controlled dietary changes render it difficult to appreciate these results. The more recent use of multi-micronutrient-fortified biscuits by Nga and co-workers [47,48] is a compromise between the two ends of the spectrum, allowing artificial nutrients to be delivered through food. However, this is the only group that employed this strategy and more evidence is needed to confirm its efficacy in preventing re-infection with soil-transmitted helminths.

It must be noted that nutrients often have interactions that affect their absorption and presumably their impact on soil-transmitted helminth (re-)infection. Antagonistic interactions were observed in two studies pertaining to *A. lumbricoides* infection [39,45]. However, in one of these studies [39], vitamin A and zinc were also shown to work synergistically and better than placebo or taking the supplements individually in reducing the mean duration of *A. lumbricoides* infection and the incidence of diarrhoea. In order to fully exploit the potential of nutritional supplementation for reducing (re-)infection with soil-transmitted helminths, the careful identification of synergistic combinations of supplements is thus required.

On a more fundamental note, it is currently unclear how long it will take for a reliable source of nutrition to become utilised for the building up of immune defenses and not for catching up on retarded growth. Many of the populations from the studies reviewed here have suffered from chronic undernutrition and high prevalence of soil-transmitted helminths and other infectious diseases, and hence, the treatment, nutritional supplementation given and the observation period might not be adequate or sufficient for the body to recover from the accumulated growth retardation, to wipe out infections and to strengthen the immune system at the same time. Therefore, more rigorous chemotherapies, such as triple-dose regimens or combination therapies [56-60] repeated over an extended period of time as well as continuous nutritional supplementation or markedly improved food supplies both in terms of quality and quantity might be needed. Such a situation might be more suitable for an accurate test of the impact of nutritional supplementation on (re-)infection with soil-transmitted helminths.

## Conclusions

We conclude that the current evidence-base for the effect of nutrition on (re-)infection with soil-transmitted helminths is weak and of low quality. Hence, no guidelines on nutrition management with or without preventive chemotherapy can be derived from it. In order to generate the required evidence for policy makers to base their recommendations on, future studies should focus on having a rigorous study design, and consider whole foods, the entire diet as well as combination supplementation as intervention tools. Making sure that the body has sufficient time to recover from undernutrition and soil-transmitted helminth infection before the final evaluation of the intervention is another requirement. Finally, it is important to realise that multi-pronged approaches are probably necessary to prevent and control the negative effects of soil-transmitted helminth infections, including anthelminthic drugs, safe water and sanitation, proper hygiene habits and, possibly, improved nutrition.

## Competing interests

The authors declare that they have no competing interests.

## Authors’ contributions

PY conceived the study, extracted, analysed and interpreted the data and prepared the manuscript. JU and JH interpreted the data and revised the manuscript. PS conceived the study, interpreted the data and revised the manuscript. All authors read and approved the final version of the manuscript.

## Supplementary Material

Additional file 1: Table S1 Detailed search strategies for this systematic review. Click here for file

Additional file 2: Table S2 List of the eight studies excluded from this systematic review. Click here for file

Additional file 3: Table S3 Detailed summary of primary and secondary outcomes of the 15 studies retained for inclusion in the systematic review. Click here for file

Additional file 4: Table S4 PRISMA checklist. Click here for file
