# Supplementary material for: Influence of nutrition on infection and re-infection with soil-transmitted helminths: a systematic review
Source: Parasit Vectors. 2014 May 19;7:229. doi: 10.1186/1756-3305-7-229 (PMC4032457; doi:10.1186/1756-3305-7-229)
Supplement: Additional file 1: Table S1 — Detailed search strategies for this systematic review. [file 1756-3305-7-229-S1.pdf]

Databases	Search sets
**1) Pubmed/Medline**<br>**2) Embase**<br>**3) Cochrane Library**<br>**4) Cochrane Central Register of Controlled Trials**<br>**5) Virtual Health Library**	▪ Reinfection AND nutrition<br>▪ Reinfection AND undernutrition<br>▪ Reinfection AND malnutrition<br>▪ Reinfection AND iron<br>▪ Reinfection AND zinc<br>▪ Reinfection AND vitamin<br>▪ Reinfection AND nutritional supplementation<br>▪ Multiparasitism AND nutrition<br>▪ Polyparasitism AND nutrition<br>▪ Infection intensity AND nutrition<br>▪ Soil-transmitted helminths AND nutrition<br>▪ Soil-transmitted helminths AND reinfection<br>▪ Soil-transmitted helminths AND undernutrition<br>▪ Soil-transmitted helminths AND micronutrient supplementation<br>▪ Hookworm AND nutritional supplementation<br>▪ *Trichuris* AND nutritional supplementation<br>▪ *Ascaris* AND nutritional supplementation<br>▪ *Strongyloides* AND nutritional supplmentation
**6) Science Direct**	All the above except:<br>▪ Infection intensity AND nutrition
**7) VIP Information**<br>**8) China National Knowledge Infrastructure**	▪ 土源性蠕虫 (Soil-transmitted helminths)<br>▪ 蛔虫感染 (Ascariasis)<br>▪ 鞭虫感染 (Trichuriasis)<br>▪ 钩虫感染 (Ancylostomiasis)
