# Supplementary material for: Influence of nutrition on infection and re-infection with soil-transmitted helminths: a systematic review
Source: Parasit Vectors. 2014 May 19;7:229. doi: 10.1186/1756-3305-7-229 (PMC4032457; doi:10.1186/1756-3305-7-229)
Supplement: Additional file 2: Table S2 — List of the eight studies excluded from this systematic review. [file 1756-3305-7-229-S2.pdf]

**Study**	**Reasons for exclusion**
Ahmann *et al.* 1933	Case series of 3 individuals
Bundy *et al.* 1987	A review on the mechanisms of interactions between helminths and host malnutrition
Figaro-Fletcher *et al.* 1988	Unable to obtain full text; only the abstract was available
Quihui-Cota *et al.* 2004	A cross-sectional study which is unable to demonstrate causality
Hughes *et al.* 2006	Focuses on general interactions between malnutrition and immune impairment without being specific for soil-transmitted helminth infection
Koski *et al.* 2001	A review on the effects of nutritional deficiencies on gastrointestinal nematodes of humans, livestock and laboratory rodents
Neumann *et al.* 1975	Focuses on general immunologic responses in malnourished children without being specific for soil-transmitted helminth infection
Tripathy *et al.* 1971	Case series of 12 individuals
