# Supplementary material for: Influence of nutrition on infection and re-infection with soil-transmitted helminths: a systematic review
Source: Parasit Vectors. 2014 May 19;7:229. doi: 10.1186/1756-3305-7-229 (PMC4032457; doi:10.1186/1756-3305-7-229)
Supplement: Additional file 3: Table S3 — Detailed summary of primary and secondary outcomes of the 15 studies retained for inclusion in the systematic review. [file 1756-3305-7-229-S3.pdf]

Study	Baseline Prevalence/ Infection Intensity	Outcomes
1) Nga *et al.* 2011	Infection intensity:<br>i) 'Fortified biscuits and albendazole': 10,032 EPG for *A. lumbricoides*; 264 EPG for *T. trichiura*; 852 PEG for hookworm<br>ii) 'Albendazole alone': 10,008 EPG for *A. lumbricoides*; 312 EPG for *T. trichiura*; 864 EPG for hookworm	**Primary:** i) **Significant**[a] **difference in infection intensity (EPG) reduction observed for *A. lumbricoides* after 4 months**<br>*Differences in infection intensity between 4 months and baseline for children taking 'fortified biscuits and albendazole' versus 'albendazole alone'*: for *A.* lumbricoides: -7,728 EPG *versus* -4,656 EPG. For *T. trichiura*: -72 EPG *versus* -48 EPG and for hookworm: -672 EPG versus -552 EPG, but differences for *T. trichiura* and hookworm were not statistically significant.<br>**Secondary:** Children receiving fortified biscuits i) had their mid-upper arm circumference slightly improved (+0.082 cm) and ii) scored higher (+0.34) on the digit span forward cognitive test. These improvements were statistically significant
2) Nga *et al.* 2009	Prevalence:<br>i) 'Fortified biscuits and albendazole': 68% for *A. lumbricoides*; 58% for *T. trichiura*; 7% for hookworm<br>ii) 'Albendazole alone': 65% for *A. lumbricoides*; 53% for *T. trichiura*; 6% for hookworm	**Primary:** i) **Significant difference in prevalence reduction observed for *A. lumbricoides* and *T. trichiura* after 4 months**<br>*Differences in prevalence between 4 months and baseline for children taking 'fortified biscuits and albendazole' versus 'albendazole alone'*: for *A.* lumbricoides: -25% *versus* -9% and for *T. trichiura*: -19% *versus* -4%. For hookworm: -2% *versus* 0% but differences for hookworm were not statistically significant<br>**Secondary:** Fortified biscuits significantly reduced the odds of i) anaemia and ii) deficiencies of zinc and iodine by 44%, 48% and 47%, respectively
3) Nchito *et al.*	Prevalence of *A. lumbricoides*:	**Primary:** i) **No significant difference in re-infection rate and infection intensity of *A. lumbricoides* between the different intervention groups**

2009	i) ‘Iron only’: 42%; ‘multi-micronutrients only’: 44%; ‘placebo’: 45%; ‘iron with multi-micronutrients’: 44%<br>Infection intensity of *A. lumbricoides*:<br>i) ‘Iron only’: 3135 EPG; ‘multi-micronutrients only’: 1645 EPG; ‘placebo’: 2655 EPG; ‘iron with multi-micronutrients’: 2972 EPG	*Differences in prevalence between 6 months and baseline*: -21% in ‘iron only’ *versus* -18% in ‘multi-micronutrients only’, -12% in ‘placebo’ and -6% in ‘iron with multi-micronutrients’. In all intervention arms, prevalences were back to baseline levels at 10 months<br>*Differences in infection intensity as compared to baseline*: at 6 months, -1,726 EPG in ‘iron only’ *versus* -1,369 EPG in ‘iron with multi-micronutrients’, +1,501 EPG in ‘multi-micronutrients only’ and +380 EPG in ‘placebo’ At 10 months, -1,233 EPG in ‘iron only’ *versus* -9 EPG in ‘placebo’, +138 EPG in ‘iron with multi-micronutrients’ and +983 EPG in ‘multi-micronutrients only’<br>ii) **There was a significant interaction between iron and multi-micronutrient supplementation on re-infection rate at 6 months**<br>*Re-infection rate:* 22% in ‘iron taken without multi-micronutrients’ *versus* 38% in ‘iron taken with multi-micronutrients’<br>iii) **A significant dose-response relationship between the number of iron tablets taken and the reduction in infection intensity was observed at 6 months.** Children who took iron were re-infected with 50% of the infection intensity found in the placebo group. In a sub-group analysis, children who took more than 50 iron tablets were re-infected with 20% of the infection intensity found in children who took placebo tablets
4) Long *et al.* 2007	Baseline prevalence was not provided and only re-infection rates were given	**Primary:** i) ‘**Zinc alone’ significantly increased the prevalence of *A. lumbricoides* by 51% as compared to placebo. ‘**Vitamin A with zinc’ decreased the prevalence by 1% and ‘vitamin A alone’ increased the prevalence by 18% as compared to placebo. Both changes were not statistically significant<br>**Secondary:** i) A combination of vitamin A and zinc significantly reduced

		the mean duration of *A. lumbricoides* infection (3.6 days) as compared to placebo (5.5 days)<br>ii) A combination of vitamin A and zinc significantly reduced the incidence of *A. lumbricoides*-associated diarrhoea by 73%
5) Long *et al.* 2006	Baseline infection characteristics not provided	**Secondary:** i) In the event of an *A. lumbricoides* infection, vitamin A supplemented children had increased interleukin 4 (IL-4) levels compared to un-supplemented children (odds ratio = 12.06)
6) Olsen *et al.* 2003	Baseline infection characteristics not provided	**Primary:** i) **No significant difference in re-infection rate and infection intensity throughout the 11 months**<br>*Differences in prevalence between 11 months and baseline for children taking 'multi-micronutrients'* versus *'placebo'*: for *A. lumbricoides*: -1% *versus* -0.2%; for *T. trichiura*: -26% versus -27%; for hookworm: -37% versus -35%<br>*Differences in infection intensity between 11 months and baseline for children taking 'multi-micronutrients'* versus *'placebo'*: for *A. lumbricoides*: -0.5 EPG *versus* -0.3 EPG; for *T. trichiura*: -4 EPG for both groups; for hookworm: -7 EPG *versus* -6 EPG
7) Olsen *et al.* 2000	Prevalence in children:<br>i) 'Iron': 27% for *A. lumbricoides*; 41% for *T. trichiura*; 62% for hookworm<br>ii) 'Placebo': 27% for *A. lumbricoides*; 38% for *T. trichiura*; 58% for hookworm<br>Infection intensity in children:<br>i) 'Iron': 2,630 EPG for *A. lumbricoides*; 25 EPG for *T. trichiura*; 50 EPG for	**Primary:** i) **No significant difference in re-infection rate and infection intensity in children after 12 months.**<br>*Differences in prevalence between 12 months and baseline for children taking 'iron' versus 'placebo'*: for *A. lumbricoides:* +15% for both groups; for *T. trichiura:* -11% *versus* -6%; for hookworm: -30% versus -25%<br>*Differences in infection intensity between 12 months and baseline for children taking 'iron'* versus *'placebo'*: for *A. lumbricoides*: +1,115 EPG *versus* -1,710 EPG; for *T. trichiura*: +13 EPG *versus* -15 EPG; for

	hookworm<br>ii) 'Placebo': 7,615 EPG for *A. lumbricoides*; 45 EPG for *T. trichiura*; 35 EPG for hookworm<br>Prevalence in adults:<br>i) 'Iron': 8% for *A. lumbricoides*; 17% for *T. trichiura*; 72% for hookworm<br>ii) 'Placebo': 6% for *A. lumbricoides*; 19% for *T. trichiura*; 67% for hookworm<br>Infection intensity in adults:<br>i) 'Iron': 1,395 EPG for *A. lumbricoides*; 15 EPG for *T. trichiura*; 75 EPG for hookworm<br>ii) 'Placebo': 4,205 EPG for *A. lumbricoides*; 20 EPG for *T. trichiura*; 88 EPG for hookworm	hookworm: -10 EPG *versus* +10 EPG<br>ii) **Significant difference in re-infection rate observed in adults at 4 or 12 months**<br>*Differences in prevalence as compared to baseline for adults taking 'iron'* versus *'placebo'*: at 4 months for hookworm: -61% versus -67%. Re-infection rates for *A. lumbricoides* and *T. trichiura* at 4 months were not reported. At 12 months, for *A. lumbricoides*: + 9% *versus* +26%; for *T. trichiura*: -11% *versus* + 2%; for hookworm: -37% for both groups but not statistically significant<br>iii) **No significant difference in infection intensity in adults at 12 months.**<br>*Differences in infection intensity between 12 months and baseline for adults taking 'iron' versus 'placebo'*: for *A. lumbricoides*: -1,085 EPG *versus* -3,795 EPG; for *T. trichiura:* +8 EPG *versus* +5 EPG; for hookworm: -45 EPG *versus* +58 EPG
8) Grazioso *et al.* 1993	Prevalence (stratification of helminthic species not done):<br>i) 'Zinc': 43%; 'placebo': 41%	**Primary:** i) **No significant difference in re-infection rate and infection intensity at the end of 120-150 days**<br>*Difference in prevalence between end of 120-150 days and baseline for children taking 'zinc' versus 'placebo'*: -65% versus -66%. Specific prevalences of *A. lumbricoides* and *T. trichiura* after the mebendazole therapy were not mentioned. Actual values of infection intensity were also not reported

9) Halpenny *et al.*2013	Cycle 1:<br><br>i) Prevalence of whole cohort:<br>*A. lumbricoides*: 20%; Hookworm: 5%; *T. trichiura*: 1%<br><br>ii) Infection intensity of whole cohort:<br>*A. lumbricoides*: 3,000 EPG; Hookworm: 250 EPG; *T. trichiura*: 50 EPG<br><br>Cycle 2:<br><br>i) Prevalence of whole cohort:<br>*A. lumbricoides*: 18%; Hookworm: 23%; *T. trichiura*: 10%<br><br>ii) Infection intensity of whole cohort:<br>*A. lumbricoides*: 10,000 EPG; Hookworm: 200 EPG; *T. trichiura*: 450 EPG	**Primary:** i) Children with higher height-for-age (HAZ) score have hookworm infection intensity 0.49 times that of their peers with lower HAZ score at the end of cycle 2. This was statistically significant<br><br>ii) Children with higher HAZ score have *A. lumbricoides* infection intensity 0.15 times that of their peers with lower HAZ score at the end of cycle 1. This was statistically significant
10) Hesham Al-Mekhlafi *et al.* 2008	Prevalence of whole cohort:<br><br>i) *A. lumbricoides*: 66%; Hookworm: 11%; *T. trichiura*: 98%	**Primary:** i) **Three months after de-worming with albendazole, stunted children had a higher re-infection rate (61%) with soil-transmitted helminths (stratification of species not done) than non-stunted children (40%) only in the univariate analysis.** This significant difference was lost in the multivariate analysis. Also at 3 months, children with underweight *versus* non-underweight have re-infection rates of 51% *versus* 43% but this was not statistically significant<br>*Re-infection rates at 6 months*: stunted children *versus* non-stunted children were 88% *versus* 73%, while underweight *versus* non-underweight were 86% *versus* 75%. However, these observations were not statistically significant

11) Payne *et al.* 2007	Prevalence of *A. lumbricoides*:<br>i) 'Vitamin A': 71%; 'non-Vitamin A': 86%<br>ii) 'Stunted': 84%; 'non-stunted': 77%<br>Infection intensity of *A. lumbricoides*:<br>i) 'Vitamin A': 949 EPG; 'non-Vitamin A': 3416 EPG<br>ii) 'Stunted': 2956 EPG; 'non-stunted': 2026 EPG	**Primary:** i) **Vitamin A supplemented children had significantly lower (-3.6 EPG) infection intensity at 3 months as compared to non-supplemented ones.** However, infection intensity for supplemented children was already significantly lower (-6.3 EPG) at baseline<br>ii) **Stunted children had significantly higher *A. lumbricoides* infection intensity than non-stunted** at both 3 (+1.6 EPG) and 5 (+2.0 EPG) months, regardless of vitamin A supplementation<br>iii) In non-stunted children, prevalence and infection intensity of *A. lumbricoides* were significantly lower in supplemented children (13% and 1 EPG) than non-supplemented ones (45% and 32 EPG) at 3 months. At 5 months, the differences were 50% and 54 EPG *versus* 55% and 147 EPG but they were not statistically significant anymore<br>iv) In stunted children, prevalence and infection intensity of *A. lumbricoides* between supplemented and non-supplemented ones were not statistically significant at 3 and 5 months. At 3 months: 38% and 19 EPG *versus* 50% and 39 EPG. At 5 months, 73% and 544 EPG *versus* 70% and 102 EPG<br>v) In stunted children, infection intensity of *A. lumbricoides* at 3 months was significantly lower (6 EPG) in children who received vitamin A within 6 weeks of de-worming as compared to children who received vitamin A between 6 and 12 weeks before de-worming (122 EPG)<br>vi) The 3-month re-infection rate of *A. lumbricoides* increased significantly as the interval between supplementation and de-worming increased. Exact figures are not reported
12) Saldiva *et al.* 2002	Baseline infection characteristics not provided	**Primary:** i) After 1 year of follow-up, 38% of the undernourished children were re-infected with helminths, in particular *A. lumbricoides*

		and *T. trichiura*, while only 25% of eutrophic children were re-infected. However, this statistically significant difference was lost once maternal literacy and per capita incoming rate were controlled for
13) Hagel *et al.* 1999	Baseline infection characteristics not provided	**Primary:** i) At 8 months after the end of the 12-month treatment period, children ≤10th percentile for height and weight/age (68% and 87%, respectively) showed significantly higher re-infection rates with *A. lumbricoides* than children >10th percentile (32% and 13%, respectively). However, this observation could be confounded by socio-economic factors (not accounted for in the analysis), as there were a significantly higher proportion of children at or below the 10th percentile for height or weight/age in extreme poverty as compared to children in critical poverty
14) Kightlinger *et al.* 1996	Infection intensity of 2765 EPG for the whole cohort	**Primary:** i) After 12 months, the best-nourished children had lower *A. lumbricoides* egg counts than children with reduced growth indicators, but these differences were not statistically significant<br>*Egg counts for best-nourished* versus *under-nourished children:* in terms of weight-for-age, 1,995 EPG *versus* 3,162–6,310 EPG and in terms of height-for-age, 3,162 EPG *versus* 3,981–5,012 EPG<br>iii) Difference in worm burden (geometric mean number of worms per child) among children with normal growth (12.5) *versus* stunted and underweight (11.5) *versus* stunted, underweight and wasted (16.0) was not significant.
15) Hagel *et al.* 1995	Baseline infection characteristics not provided	**Secondary:** i) **No significant change in anti-*Ascaris* IgE levels was observed in undernourished children, while levels in well-nourished children increased significantly**<br>*Differences between 12 months and baseline for under-nourished* versus

	well-nourished: in terms of weight-for-age, -0.10 pru/mL *versus* +0.55 pru/mL; in terms of height-for-age, -0.05 pru/mL *versus* +0.65 pru/mL
